# Modified balloons to prepare severely calcified coronary lesions before stent implantation: a systematic review and meta-analysis of randomized trials

**DOI:** 10.1007/s00392-023-02324-y

**Published:** 2023-11-06

**Authors:** Maria Scalamogna, Constantin Kuna, Felix Voll, Alp Aytekin, Shqipdona Lahu, Thorsten Kessler, Sebastian Kufner, Tobias Rheude, Hendrik B. Sager, Erion Xhepa, Jens Wiebe, Michael Joner, Gjin Ndrepepa, Adnan Kastrati, Salvatore Cassese

**Affiliations:** 1 Klinik Für Herz- Und Kreislauferkrankungen, Deutsches Herzzentrum München, Technische Universität München, Lazarettstrasse, 36, Munich, Germany; 2 Department of Advanced Biomedical Sciences, University of Naples Federico II, Naples, Italy; 3 DZHK (German Centre for Cardiovascular Research), Partner Site Munich Heart Alliance, Munich, Germany

**Keywords:** Coronary artery disease, Meta-analysis, Modified balloons, Stent

## Abstract

**Background:**

The performance of modified balloons (namely cutting or scoring balloons) to prepare severely calcified lesions in patients undergoing percutaneous coronary intervention (PCI) remains controversial. We investigated the clinical and imaging outcomes of patients undergoing PCI assigned to modified balloon therapy to prepare severely calcified coronary lesions before stent implantation.

**Methods:**

In this meta-analysis, we aggregated the study-level data from trials enrolling invasively treated patients who were randomly assigned to modified balloon or control therapy to prepare severely calcified lesions before stenting. The primary outcome was major adverse cardiac events (MACE), including death, myocardial infarction (MI), and repeat revascularization. The secondary outcomes included the individual components of the primary outcome, coronary perforation and final minimal stent area (MSA) as measured by intracoronary imaging.

**Results:**

A total of 648 participants in six trials were allocated to modified balloon therapy (*n* = 335) or control therapy (semi-compliant, non-compliant, or super high-pressure balloon, *n* = 313). The median follow-up was 11 months. Overall, MACE occurred in 8.96% of patients assigned to a modified balloon and 12.78% of patients assigned to control therapy [risk ratio = 0.70, 95% confidence interval (CI) 0.35–1.39; *P* = 0.24]. There was a significant treatment effect-by-modified balloon type interaction for the outcome MACE in patients assigned to cutting balloon compared with control therapy [RR = 0.40 (0.28–0.56), P for interaction (*P*_int_) < 0.001]. Patients treated with a modified balloon compared with control therapy showed neither a significant difference for the other clinical outcomes nor for final MSA [standardized mean difference = 0.67 (− 0.71, 2.06); *P* = 0.26].

**Conclusions:**

In patients treated with PCI for severely calcific coronary artery disease a strategy of lesion preparation with a modified balloon before stenting does not improve clinical or imaging outcomes compared with control therapy. The different performance of cutting and scoring balloons warrants further investigation.

**Graphical Abstract:**

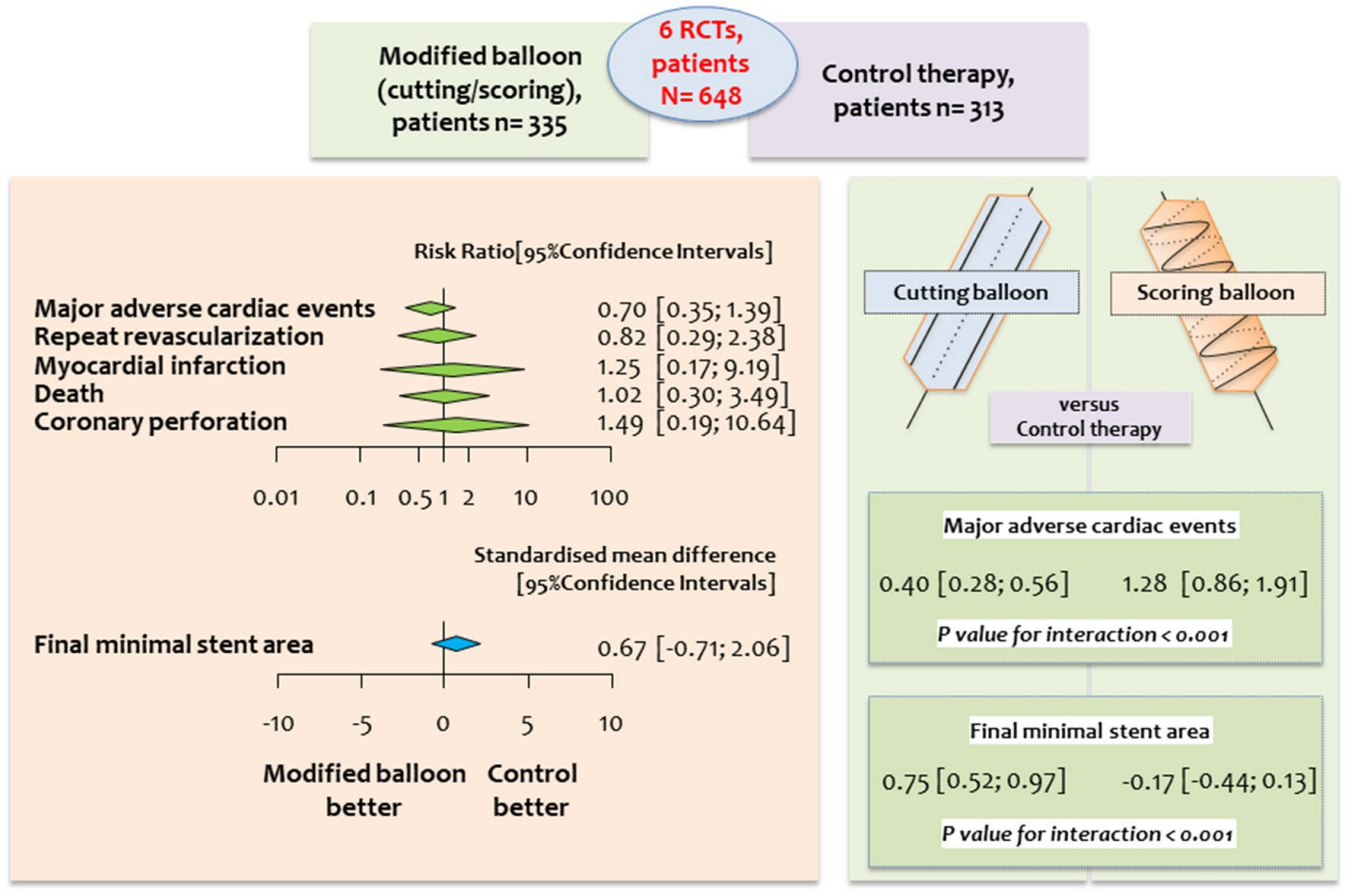

**Supplementary Information:**

The online version contains supplementary material available at 10.1007/s00392-023-02324-y.

## Introduction

Percutaneous coronary intervention (PCI) in patients with calcific coronary artery disease (CAD) represents a great challenge even with contemporary high-performance percutaneous technologies [1]. Vascular calcification reduces vessel compliance, increases the risk of peri-procedural complications, and may interfere with the mechanical behaviour of stent platforms over the long term [2]. Lesion preparation before stent implantation is a prerequisite in patients with severely calcified coronary lesions, to minimize underexpansion or structural damage to stent platforms and to enhance the uptake of anti-proliferative drugs from the stent surface into the vessel wall [3].

Among lesion preparation and calcium modification strategies, non-compliant balloons remain the first-line therapy in PCI patients amenable to stent implantation. In general, calcified coronary lesions are prepared by inflating a standard non-compliant balloon to high pressure before stenting. For certain lesions, such as those with severe calcifications, high-pressure non-compliant balloon inflation might be insufficient to achieve adequate vessel preparation before stent implantation. For this reason, alternative balloon-based lesion preparation strategies, including modified balloons (namely cutting or scoring balloons), have been tested in patients with calcific CAD [4].

Cutting balloons are semi-compliant balloons with microsurgical blades mounted longitudinally along the balloon surface, while scoring balloons are semi-compliant balloons with a wire system surrounding the balloon surface [1]. Both technologies create controlled incisions in calcified plaque at low inflation pressures to potentially increase vessel compliance, and thereby final stent expansion. Previous data regarding the performance of modified balloons to prepare severely calcified coronary lesions before stent implantation have been controversial [5, 6]. Therefore, this meta-analysis investigates the clinical and imaging outcomes of PCI patients randomly assigned to modified balloons to prepare severely calcified coronary lesions before stent implantation.

## Methods

### Data sources and searches

Major scientific databases, scientific abstracts of major cardiovascular conferences, and clinical trial registration websites were searched from the start of each database through May 2023 for randomized trials investigating patients undergoing PCI using modified balloon versus control therapy (semi-compliant, non-compliant, or super high pressure balloon) to prepare severely calcified coronary lesions before stenting. We extrapolated further citations by reviewing the reference lists in all eligible studies. Search terms included the keywords and the corresponding Medical Subject Headings for: “balloon angioplasty”, “cutting balloon” or “scoring balloon”, “calcium” or “calcified lesions”, “percutaneous coronary intervention”, “stent”, “trial”, and “randomized trial”. Inclusion criteria were: (1) lesion preparation with modified balloon versus control therapy; (2) randomized design; (3) intracoronary imaging after stent implantation; (4) ≥ 30-day clinical follow-up. Comparisons other than modified balloon versus control therapy were ineligible. The upfront or bailout use of rotational atherectomy (RA) as complementary lesion preparation in one or both treatment groups was not an exclusion criterion.

### Study selection

Two investigators (MS and CK) independently assessed publications for eligibility at the title and/or abstract level. A third investigator (FV) helped resolve possible divergences. If the studies met the inclusion criteria, they were included in further analysis.

### Data extraction, quality assessment and outcome variables

Trial-level data concerning the overall number of patients, mean age, males’ proportion, the proportion of patients with diabetes mellitus, arterial hypertension or acute coronary syndrome (ACS) on admission, treated vessel, reference vessel diameter (RVD), lesion length, diameter stenosis (DS), and calcium arch degree as assessed by intracoronary imaging before PCI were extracted from each trial. The risk of bias was evaluated independently for each study by the same investigators, in accordance with The Cochrane risk-of-bias tool for randomized trials version 2 (RoB2) to assess the quality of included trials [7]. We did not assign composite quality scores [8].

The primary outcome of this analysis was major adverse cardiac events (MACE), a composite outcome including (but not limited to) death, myocardial infarction (MI), and repeat revascularization. The secondary outcomes included the individual components of the primary outcome, coronary perforation and final minimal stent area (MSA) as measured by intracoronary imaging. We considered all endpoints occurring up to the maximum follow-up duration available in the intention-to-treat population (unless otherwise specified) and as per definitions reported in the original protocols.

### Data synthesis and analysis

Means for continuous variables and proportions for categorical variables were displayed as exploratory analyses for baseline features of participants enrolled in each included study. The weighted median follow-up duration was calculated based on the sample size of each individual study. Risk ratios (RRs) or bias-corrected standardized mean difference (SMD) with 95% confidence intervals (CI) and *P*-value < 0.05 were used to compare outcomes of interest between treatment groups. Study-level risk estimates were pooled using the Mantel–Haenszel random-effect model or the inverse variance weighting with the Hartung–Knapp adjustment. To account for imbalances in follow-up duration among included studies, we calculated random-effects incident rate ratios (IRR) with pertinent 95% CI for the primary outcome. Heterogeneity between trials was quantified using the *I*^2^ statistic accompanied by a Chi-square test: *I*^2^ values approaching 25%, 50%, and 75% indicated low, moderate, and high heterogeneity, respectively [9]. In addition, we estimated the between-study variance using the Paule–Mandel or DerSimonian and Laird estimator for tau^2^ for each outcome. For the primary outcome, we displayed the 95% prediction interval of the pooled estimate [10]. Treatment effect was not assessed in trials in which no events were reported within-groups. The possibility of small study effects due to publication bias or other biases was examined for the primary outcome by means of visual inspection of funnel plots of the RRs of individual trials against their standard errors. A linear regression test for funnel plot asymmetry and an influence analysis, in which meta-analysis estimates are computed omitting one study at a time, were performed for the primary outcome.

Using a Chi-square test for treatment-by-subgroup interaction, we tested whether the predominant use of either cutting balloon or scoring balloon in the experimental arm and the upfront use of RA was associated with a modification of the treatment effect for the outcome MACE. The same statistical method served to explore whether there was a treatment-by-coronary imaging interaction (IVUS versus OCT) for the outcome final MSA. In addition, to further account for the different treatment strategies pooled in this study, we performed a frequentist network meta-analysis for the outcome MACE according to Rücker et al. [11] (package *netmeta*), providing a treatment ranking based on the *P*-scores, which measure the mean extent of certainty that a treatment is better than the competing treatments. Finally, a random-effects meta-regression analysis assessed the modification of the treatment effect for the primary outcome based on mean age, proportions according to male sex, diabetes mellitus, arterial hypertension or ACS on admission, vessel treated (left anterior descending artery versus other), lesion length, RVD, DS, and calcium arch degree at intracoronary pre-PCI imaging. We calculated the power of our meta-analysis to detect a 50% relative risk difference for main outcomes with modified-balloon conditional on the observed precision of the pooled estimate [12]. We set the 50% threshold as a benchmark because it corresponds to the predominant assumption of superiority supporting the power of contemporary clinical trials. This study was reported in compliance with the Preferred Reporting Items for Systematic reviews and Meta-Analyses (PRISMA) statement (Supplemental Table 1) [13]. All analyses were performed using the package *meta* and *metafor* in R (*version 4.1.3; R Foundation for Statistical Computing, Vienna, Austria*). No extramural funding was used to support this work. Ethical approval was not required for this study. This study is registered with PROSPERO under number CRD42022360819.

## Results

### Eligible studies

The flow diagram for the trial selection process is shown in Supplemental Fig. 1. After application of the inclusion/exclusion criteria, 6 trials, all published as full-length manuscripts [6, 14–18], were included in the meta-analysis. No disagreements required a solution by a third reviewer. In the selected trials, a total of 648 patients were randomly allocated to a modified balloon (*n* = 335) or control therapy (*n* = 313). The main characteristics of the included trials are shown in Supplemental Table 2. All trials but two [6, 17] had a multicentre design and included patients with severely calcified obstructive chronic/stable or unstable CAD amenable to coronary stenting. Three studies were conducted in China and three in Europe [15, 16, 18]. Two trials evaluated the performance of a cutting balloon versus a non-compliant balloon [6, 18], two trials compared a cutting balloon versus a semi-compliant or a non-compliant balloon after upfront RA [14, 17], one trial compared a scoring balloon versus a super high-pressure balloon [16]. The PREPARE-CALC trial compared a scoring or a cutting balloon versus upfront RA followed by non-compliant balloon dilation [15]. As the overall proportion of patients assigned to a cutting balloon in this trial was relatively low (3.3%), we considered this trial as belonging to the scoring balloon subgroup. In the modified balloon group, the cutting balloon platforms studied were the Flextome or the Wolverine (both Boston Scientific, Marlborough, MA, USA), whilst the scoring balloon platforms studied were the NSE Alpha (B. Braun, Melsungen, Germany), AngioSculpt (Spectranetics Corporation, Fremont, CA, USA) or ScoreFlex (OrbusNeich, Hong Kong, China). Four trials investigating cutting balloon versus control therapy, except one [6] suggested downsizing the study device by 0.5 mm lower than the RVD. In one trial [18], the study design recommended high-pressure inflation of the cutting balloon. In three trials [14, 15, 17], RA was performed using the Rotablator (Boston Scientific, Marlborough, MA, USA) with a burr-to-artery ratio between 0.5 and 0.7 and a rotational speed between 140.000 and 180.000 revolutions per minute (rpm). All patients received adjunctive therapies for acute or chronic CAD and were treated with standard-of-care PCI and drug-eluting stent implantation.

Intracoronary imaging data after stenting were available in 294 of 335 patients in the modified balloon group and in 272 of 313 patients in the control therapy group. Four trials performed routine IVUS investigation at baseline and after stent implantation [6, 14, 17, 18]. In these trials, the imaging systems used were Atlantis SR Pro or OptiCross coronary imaging catheters (both Boston Scientific, Marlborough, MA, USA) or Eagle Eye Gold VOLCANO S5 Imaging System (VOLCANO Corporation, San Diego, CA, USA). In one trial [18], patients with angiographic evidence of severe calcified coronary lesions were randomized only if the calcium arch degree >100 at baseline IVUS. Two trials performed a mandatory OCT investigation after stent implantation [15, 16]. In these trials, OCT acquisitions were performed with commercially available tools (ILUMIEN OPTIS system and Dragonfly OPTIS imaging catheter, both Abbott Vascular, Santa Clara, CA, USA) according to predefined standard operating procedures.

The baseline characteristics of the patients included in the original trials are shown in Table 1. Two-thirds of the patients were male, the median age was 70.9 years [interquartile range (IQR) 70–72.1], one-third had diabetes and nearly 80% of them had arterial hypertension at the time of enrolment in the primary trials. About 40% of the included patients had ACS. LAD was the target vessel in more than half of the patients and the treated vessel had a RVD of 3.04 ± 0.28 mm [median 3 mm, (IQR 2.74–3.28)] and a DS of 79.2 ± 5.7% [median 80.1 mm, (IQR 78.4–83.3)]. At baseline intracoronary imaging, the mean calcium arch degree was 238.2 ± 75.9 mm [median 240 mm, (IQR 228.6–283.9)]. The weighted median follow-up available for the assessment of outcomes of interest was 11 months (mean 15 ± 8 months).

**Table 1:** The main characteristics of the patients enrolled among trials included in the study

Trial	Patients, n	Age, years	Male, %	Diabetes mellitus, %	Hypertension, %	ACS at admission, %	Target vessel LAD, %	RVD, mm	Lesion length, mm	Diameter stenosis, %	Calcium arch, degree
COPS [18]	100	70.0	81.0	31.0	80.0	6.0	60.0	3.30	23.3	80.1	240.0
Han et al. [17]	120	71.2	65.8	35.0	41.6	N/R	54.2	2.74	N/R	84.3	319.5
ISAR-CALC [16]	74	72.1	85.1	33.7	86.5	N/R	54.1	3.00	24.1	69.8	N/R
Li et al. [14]	71	70.7	70.4	73.2	77.4	81.7	60.5	2.70	30.4	78.4	283.9
PREPARE-CALC [15]	200	74.9	76.0	33.5	93.0	8.5	49.9	3.28	20.5	83.3	118.8
Tang et al. [6]	92	61.3	67.8	16.4	70.9	72.8	83.9	N/R	N/R	N/R	228.6

Overall proportions and means are reported

ACS: acute coronary syndrome; LAD: left anterior descending; N/R: not reported; RVD: reference vessel diameter

Official titles and acronyms: COPS: Cutting balloon to Optimize Predilation for Stent implantation; ISAR-CALC: Comparison of Strategies to Prepare Severely Calcified Coronary Lesions; PREPARE-CALC: Comparison of Strategies to Prepare Severely Calcified Coronary Lesions

### Clinical and imaging outcomes (graphical abstract)

All trials had sufficient statistical power for surrogate outcomes, which included imaging efficacy measures in most cases. Two trials had available outcome data for up to 24 months [16, 17]. Outcome definitions are reported in Supplemental Table 3 and the risk of bias inter-study is presented in Supplemental Fig. 2.

### Primary outcome

**Fig. 1:**
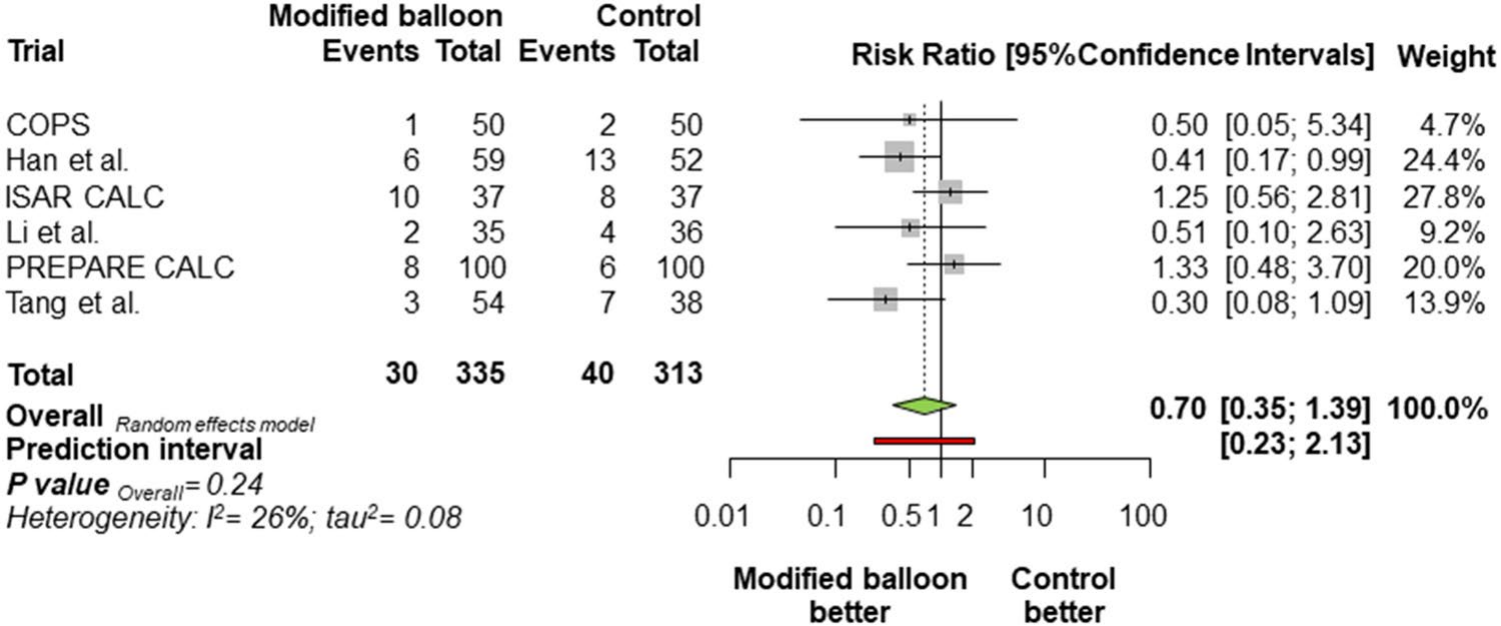
Summary of risk estimates for the primary outcome with modified balloon versus control therapy. Plot of risk ratio for major adverse cardiac events associated with modified balloon versus control therapy. The diamonds indicate the point estimate and the left and the right ends of the lines the 95% Confidence intervals. Official titles and acronyms: COPS: Cutting balloon to Optimize Predilation for Stent implantation; ISAR-CALC: Comparison of Strategies to Prepare Severely Calcified Coronary Lesions; PREPARE-CALC: Comparison of Strategies to Prepare Severely Calcified Coronary Lesions

**Fig. 2:**
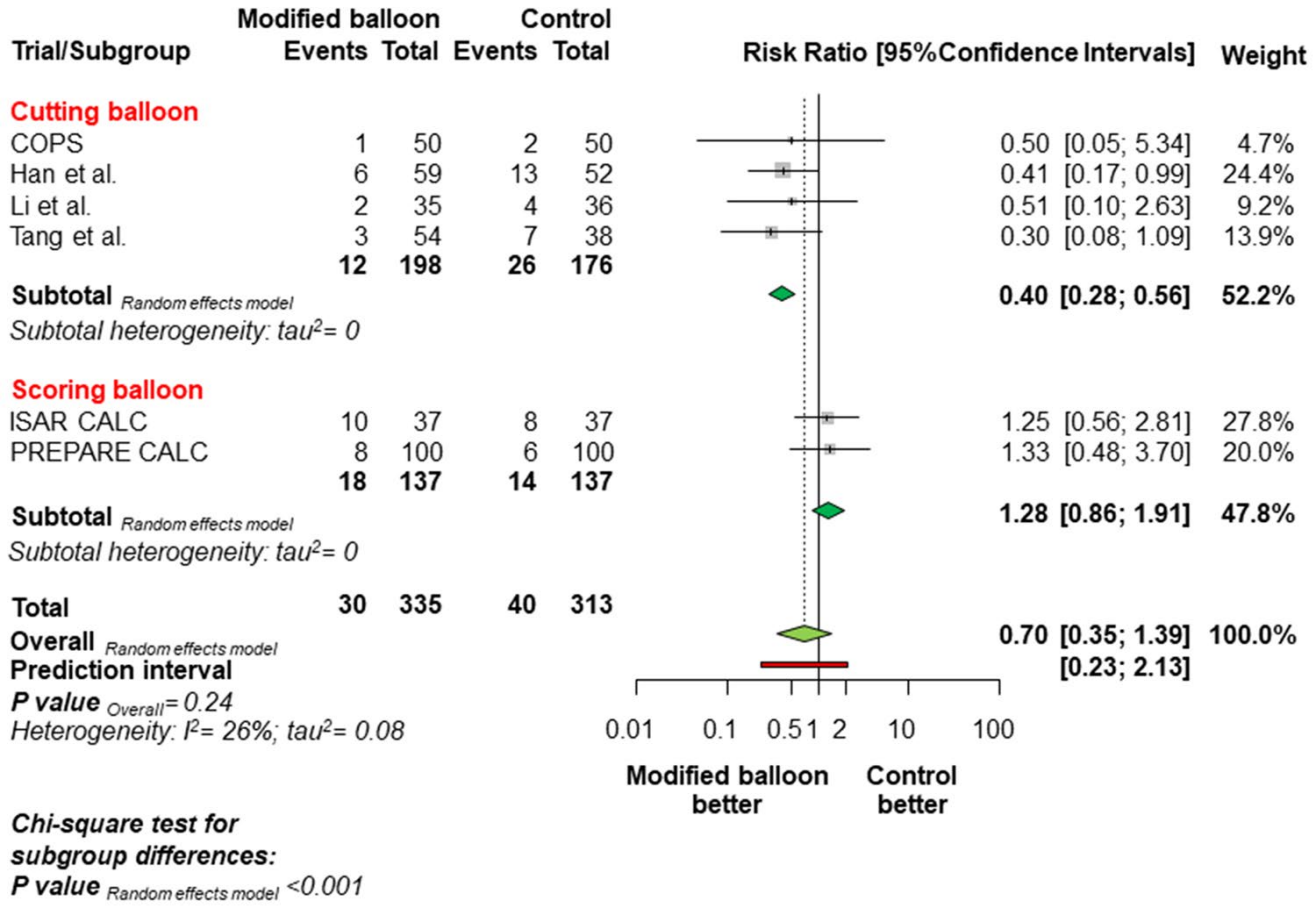
Summary of risk estimates for the primary outcome according to the type of modified balloon grouped in the experimental arm. Plot of risk ratio for major adverse cardiac events associated with modified balloon versus control therapy according to the type of modified balloon (either cutting balloon or scoring balloon). The diamonds indicates the point estimate and the left and the right ends of the lines the 95% Confidence intervals. Official titles and acronyms are as in Fig. 1

Overall, MACE occurred in 70 patients (10.80%; Fig. 1), of which 30 (8.96%) were assigned to modified balloons and 40 (12.78%) were assigned to control therapy [RR = 0.70, 95% CI 0.35–1.39; *P* = 0.24]. The random-effects meta-analysis had a power of 83.2% to detect a 50% relative risk difference for MACE associated with the use of modified balloons. The 95% prediction interval for this outcome contained the null (0.23; 2.13) and there was low to moderate heterogeneity. The different follow-up duration of the included studies did not change the direction of risk estimates for the primary outcome [IRR = 0.69 (0.34–1.39); *P* = 0.23]. Notably, there was a significant treatment-by-type of modified balloon interaction, due to a significant reduction of MACE with cutting balloon as compared with control therapy [RR = 0.40 (0.28–0.56), P for interaction (*P*_int_) < 0.001, Fig. 2]. Of note, the upfront use of RA was not associated with a significant modification of treatment effect for the primary outcome (*P*_int_ = 0.93).

### Secondary outcomes (Fig. 3A–D)

**Fig. 3:**
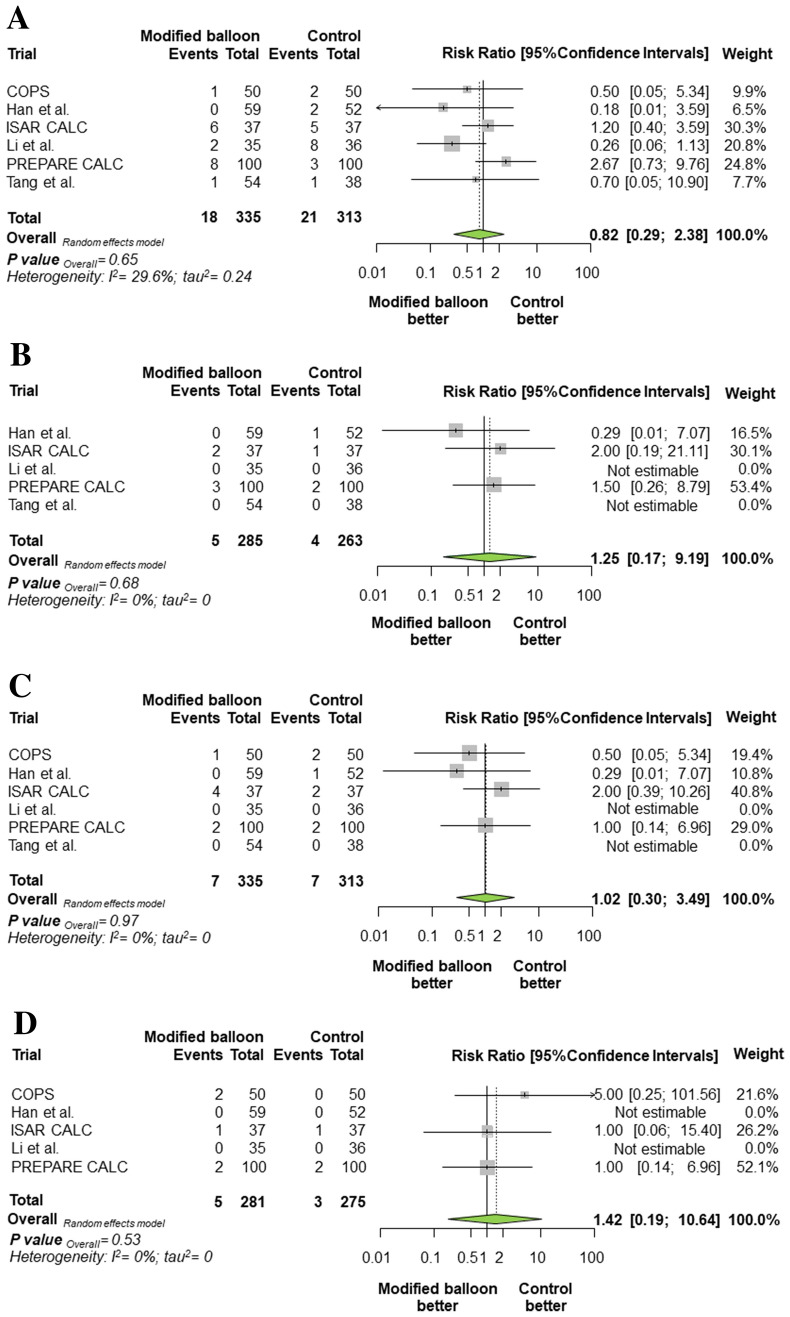
Summary of risk estimates for the secondary clinical outcomes with modified balloon versus control therapy. Plot of risk ratio for repeat revascularization (A), myocardial infarction (B), death (C), and coronary perforation (D) associated with modified balloon versus control therapy. The diamonds indicate the point estimate and the left and the right ends of the lines the 95% Confidence intervals. Official titles and acronyms as in Fig. 1

Repeat revascularization occurred in 39 patients (6.14%). The risk of repeat revascularization was not significantly different in patients assigned to modified balloon or control therapy [5.37% vs. 6.71%; RR = 0.82 (0.29–2.38), *P* = 0.65]. An exploratory analysis revealed a significant treatment effect-by-type of modified balloon interaction for this outcome favouring the use of cutting balloons (P_int_ = 0.0005).

MI occurred in 9 patients (1.64%, data available for 548 participants). The risk of MI in patients assigned modified balloon or control therapy was not significantly different [1.75% vs. 1.52%; RR = 1.25 (0.17–9.19), *P* = 0.68].

Death occurred in 14 patients (2.20%). Among patients assigned to modified balloon or control therapy, mortality was not significantly different [2.09% vs. 2.24%; RR = 1.02 (0.30–3.49), *P* = 0.97]. Cardiac death occurred in 10 patients (1.57%). The risk of cardiac death in patients assigned to modified balloon or control therapy was not significantly different [1.49% vs. 1.60%; RR = 1.04 (0.24–4.50), *P* = 0.93].

Eight patients had coronary perforations (1.80%, data available for 556 participants). The risk of coronary perforation in patients assigned to modified balloon or control therapy was not significantly different [1.78% vs. 1.09%; RR = 1.42 (0.19–10.64), *P* = 0.43].

**Fig. 4:**
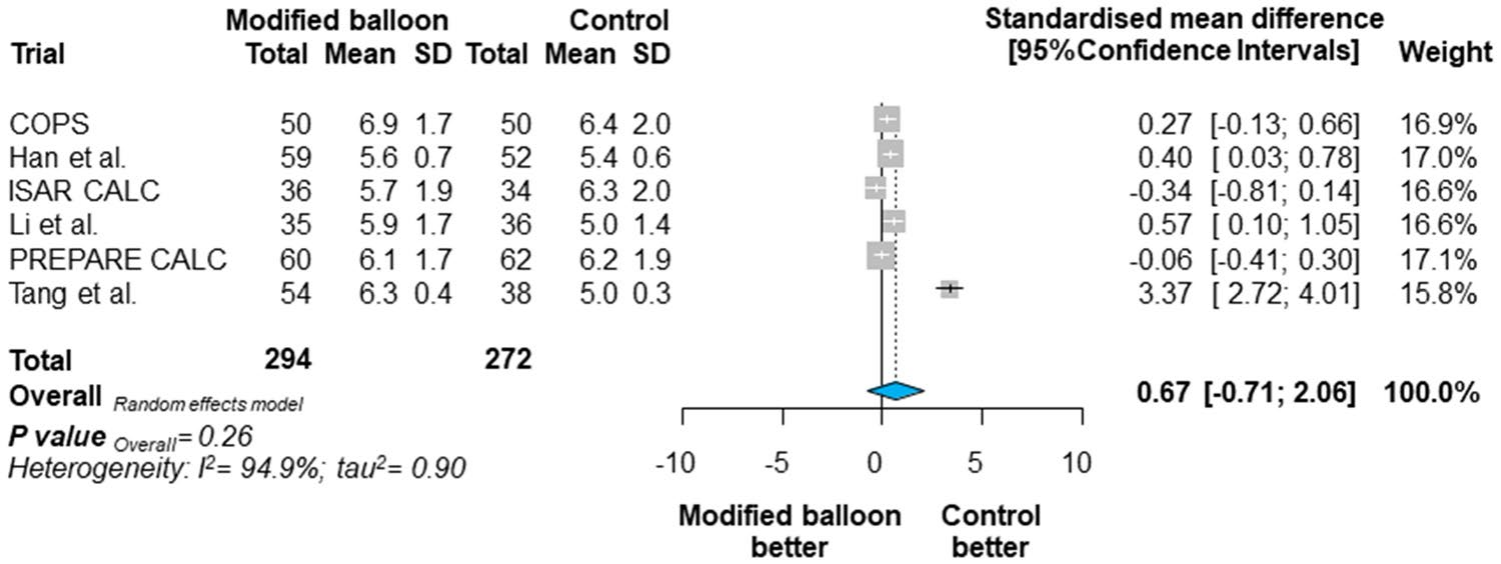
Summary of risk estimates for the final minimal stent area with modified balloon versus control therapy. Plot of standardized mean difference for final minimal stent area associated with modified balloon versus control therapy. The diamonds indicate the point estimate and the left and the right ends of the lines the [95% Confidence intervals, CIs]. Official titles and acronyms as in Fig. 1

Final MSA as assessed by intracoronary imaging was available in 566 patients (Fig. 4). Patients assigned to modified balloon versus control therapy showed no significant difference in terms of final MSA [range in mm^2^ 5.6–6.9 vs. 5.0–6.4; SMD 0.67 (− 0.71, 2.06); *P* = 0.26]. However, there was a significant treatment effect-by-type of modified balloon interaction for final MSA favouring the use of cutting balloon as compared with control therapy (*P*_int_ < 0.001), whilst there was no interaction between treatment effect and intracoronary imaging for this outcome (*P*_int_ = 0.08).

### Network, sensitivity and influence analyses

The network meta-analysis for MACE ranked cutting balloon as the best treatment option (*P*-score 0.86) followed by super high-pressure balloon (*P*-score 0.54), whilst the treatment with non-compliant balloon was ranked as the worst (*P*-score 0.32). The combination of RA and either non-compliant balloon or semi-compliant balloon showed a *P*-score of 0.51 and 0.39, respectively, whilst scoring balloon had a *P*-score of 0.35; Supplemental Table 4 and Supplemental Fig. 3). By omitting one study at a time, the direction of the summary RR for the primary outcome showed no significant modification (Supplemental Fig. 4). We excluded the potential source of bias due to a small study effect by visual inspection of contour-enhanced funnel plots of all outcomes (Supplemental Fig. 5). Although for the main outcome the absence of bias due to a small study effect was confirmed by a linear regression test of funnel plot asymmetry based on sample size (*P* = 0.45), the proficiency of this test is reduced due to the relatively small number of studies available for this analysis. Finally, the treatment effect for MACE was not dependent on age, males’ proportion, and the proportion of patients with diabetes mellitus, arterial hypertension, or ACS on admission, vessel treated, RVD, DS, and calcium arch degree (all *P* values ≥ 0.06).

## Discussion

This meta-analysis of aggregate study-level data investigated the outcomes of nearly 650 PCI patients who were randomly assigned to either modified balloon or control therapy to prepare severely calcified lesions before stent implantation. The main findings of this meta-analysis are:

- The risk of MACE in patients treated with modified balloon as compared with control therapy was not significantly different.
- Overall, the modified balloon and control therapy displayed no significant difference in terms of final MSA as measured by intracoronary imaging; however, there was a significant treatment effect-by-modified balloon type interaction owing to a larger final MSA in patients treated with cutting balloons as compared with control therapy.

To the best of our knowledge, this is the largest meta-analysis investigating the role of a modified balloon versus control therapy to prepare severely calcified coronary lesions before stent implantation. Notwithstanding the challenging anatomical subset explored in this study, both therapies demonstrated comparable safety and efficacy with a relatively low risk of adverse events and a final MSA of approaching 5.5 mm^2^ in all cases. This would lend support to a neutral effect of the modified balloon to prepare severely calcified lesions before stenting. However, the subgroup analysis suggests that the preparation of a calcified lesion with cutting balloon angioplasty reduces the risk of MACE and repeat revascularizations, and results in a greater final MSA compared with a lesion preparation with a semi-compliant or a non-compliant balloon. This advantage was not observed in patients assigned to scoring balloon therapy. The evidence of significantly different performance among modified balloons in patients with severely calcified lesions is a novel finding that merits discussion.

First, modified balloons are speciality balloons with either small cutting blades or wires that are applied in various forms to the balloon surfaces to concentrate dilation forces in specific regions of the vessel wall, thereby enhancing the luminal expansion of rigid coronary lesions [19]. Cutting and scoring balloons are mechanistically similar and are supposed to have a class effect. In particular, scoring balloon technology was developed with the intention of superseding the drawbacks of cutting balloon technology in terms of safety and deliverability [20]. In contrast, previous preclinical data suggest a different effect of cutting and scoring balloons on vessel preparation. In fact, cutting balloon technology appears to have superior efficacy in effectively penetrating the surface of the vessel wall without significant distortion of cutting elements compared with scoring balloon technology [19]. In keeping with these considerations, a previous retrospective analysis, including severely calcified lesions amenable to stent implantation, found superior acute gain and MSA with cutting balloon compared with scoring balloon [21], although the clinical correlate of this mechanical effect has never been systematically explored in a prospective fashion.

Secondly, in the current report, we found a 60% relative risk reduction in terms of MACE associated with cutting balloons versus control therapy. This result was mainly due to a 70% relative risk reduction for repeat revascularization in patients treated with a cutting balloon. Although the results of the subgroup analysis should be considered exploratory in nature, due to the lack of adequate statistical power to draw firm conclusions, the current findings are corroborated by the evidence of a larger final MSA in patients treated with cutting balloons. Final MSA is predictive of repeat revascularization and is of critical importance in patients with severely calcified lesions [22]. In the same vein, larger final MSA after lesion preparation with a cutting balloon is clinically relevant, as this result was achieved in a subset of patients with coronary calcifications involving circa two-thirds of vessel wall circumference. In fact, the calcium arch degree observed by intracoronary imaging in this study was nearly 250. This is an important aspect because it provides evidence that the treatment effect with cutting balloons is achieved despite a relatively high calcium burden, although the highest degree of coronary calcifications (≥ 270 calcium arch degree) was not included in the present analysis. As a result, the total number of adverse events remained low in absolute terms, suggesting that the population analysed for this study remains selected.

Finally, although the present meta-analysis focused on the role of modified balloon or control therapy, the upfront or bailout use of RA was permitted across treatment groups. The subgroup analysis discarded a statistical interaction between the treatment effect associated with modified balloon or control therapy and the use of RA. Consistently, the network meta-analysis performed in this study ranked cutting balloon as the best treatment option, whilst a combination with RA did not improve the ranking of non-compliant or semi-compliant balloons. Noteworthy, recent studies [23, 24] and a meta-analysis [25] suggest a possible benefit of a combination of RA and a modified balloon compared with a modified balloon or conventional balloon angioplasty as stand-alone therapies to improve outcomes in patients with severely calcified lesions undergoing PCI with stent implantation. Further studies are warranted to investigate whether the routine combination of therapies (ablative-, debulking-, balloon-based, etc.) is superior to any therapy alone to improve the preparation of severely calcified lesions amenable to stent implantation.

### Limitations

This study should be interpreted in light of some limitations. First, the meta-analysis was based on study-level data. A meta-analysis of individual participants remains the gold standard, especially for the analysis of subgroups of patients. Second, we cannot exclude that the observed differences between groups are due to the open-label design of the included trials, which may have introduced some bias in the assessment of endpoints of interest. Third, the use of different stent platforms is another limitation that needs to be mentioned. In fact, the type of stent platform has important clinical implications in patients treated with PCI for severely calcified lesions [26]. Fourth, despite we found no significant modification of risk estimates by intracoronary imaging, OCT and IVUS portend a different ability to assess calcium thickness and therefore area and volume [27]. Although this difference is unlikely to impact clinical outcomes, this limitation should be considered while interpreting the results of this analysis. Fifth, the results of this analysis do not apply to patients with clinical and anatomical features other than those presented here. Specifically, the performance of modified balloon versus control therapy in patients with more severe calcific lesions needs further investigation. In addition, the possible superior performance of cutting balloons over semi-compliant or noncompliant balloons does not imply a superiority of this technology over other balloon-based techniques (e.g., super high-pressure balloon, intravascular lithotripsy, etc.) given the lack of head-to-head comparisons. Finally, the median follow-up was 11 months; a longer follow-up would be desirable, as significant differences in longer-term follow-ups cannot be ruled out by this analysis.

## Conclusions

The present study shows that in patients undergoing PCI with severely calcified lesions, preparation with a modified balloon does not impact the risk of adverse cardiac events and final stent area compared with control therapy. The potential superior performance of the cutting balloon compared with the scoring balloon has yet to be explored in randomized trials powered for relevant clinical and imaging endpoints.

### Supplementary Information

Below is the link to the electronic supplementary material.

Supplementary file1 (DOCX 569 KB)

## Data Availability

Data available on request.

## Abbreviations

ACS: Acute coronary syndrome
CAD: Coronary artery disease
COPS: Cutting balloon to Optimize Predilation for Stent implantation
ISAR-CALC: Comparison of strategies to prepare severely calcified coronary lesions
MI: Myocardial infarction
PCI: Percutaneous coronary intervention
PREPARE-CALC: Comparison of Strategies to prepare severely calcified coronary lesions
