# Supplementary material for: Modified balloons to prepare severely calcified coronary lesions before stent implantation: a systematic review and meta-analysis of randomized trials
Source: Clin Res Cardiol. 2023 Nov 6;113(7):995–1005. doi: 10.1007/s00392-023-02324-y (PMC11219378; doi:10.1007/s00392-023-02324-y)
Supplement: Supplementary file 1 — Supplementary file1 (DOCX 569 KB) [file 392_2023_2324_MOESM1_ESM.docx]

**Appendix**

**Modified balloons to prepare severely calcified coronary lesions before stent implantation:**

**a systematic review and meta-analysis of randomized trials.**

Maria Scalamogna, MD, Constantin Kuna, MD, Felix Voll, MD, et al.

**Supplemental Table 1. PRISMA checklist**

| **Section and Topic** | **Item #** | **Checklist item** | **Page #** | |  |
| --- | --- | --- | --- | --- | --- |
| **TITLE** | | | |  | |
| Title | 1 | Identify the report as a systematic review. | 1 | |  |
| **ABSTRACT** | | | |  | |
| Abstract | 2 | See the PRISMA 2020 for Abstracts checklist. |  | |  |
| **INTRODUCTION** | | | |  | |
| Rationale | 3 | Describe the rationale for the review in the context of existing knowledge. | 5 | |  |
| Objectives | 4 | Provide an explicit statement of the objective(s) or question(s) the review addresses. | 5 | |  |
| **METHODS** | | | |  | |
| Eligibility criteria | 5 | Specify the inclusion and exclusion criteria for the review and how studies were grouped for the syntheses. | 6 | |  |
| Information sources | 6 | Specify all databases, registers, websites, organisations, reference lists and other sources searched or consulted to identify studies. Specify the date when each source was last searched or consulted. | 6 | |  |
| Search strategy | 7 | Present the full search strategies for all databases, registers and websites, including any filters and limits used. | Appendix, 4 | |  |
| Selection process | 8 | Specify the methods used to decide whether a study met the inclusion criteria of the review, including how many reviewers screened each record and each report retrieved, whether they worked independently, and if applicable, details of automation tools used in the process. | 6 | |  |
| Data collection process | 9 | Specify the methods used to collect data from reports, including how many reviewers collected data from each report, whether they worked independently, any processes for obtaining or confirming data from study investigators, and if applicable, details of automation tools used in the process. | 6 | |  |
| Data items | 10a | List and define all outcomes for which data were sought. Specify whether all results that were compatible with each outcome domain in each study were sought (e.g. for all measures, time points, analyses), and if not, the methods used to decide which results to collect. | 7 | |  |
|  | 10b | List and define all other variables for which data were sought (e.g. participant and intervention characteristics, funding sources). Describe any assumptions made about any missing or unclear information. | 6, 7 | |  |
| Study risk of bias assessment | 11 | Specify the methods used to assess risk of bias in the included studies, including details of the tool(s) used, how many reviewers assessed each study and whether they worked independently, and if applicable, details of automation tools used in the process. | 7, 8 | |  |
| Effect measures | 12 | Specify for each outcome the effect measure(s) (e.g. risk ratio, mean difference) used in the synthesis or presentation of results. | 7 | |  |
| Synthesis methods | 13a | Describe the processes used to decide which studies were eligible for each synthesis (e.g. tabulating the study intervention characteristics and comparing against the planned groups for each synthesis (item #5)). | 8 | |  |
|  | 13b | Describe any methods required to prepare the data for presentation or synthesis, such as handling of missing summary statistics, or data conversions. | 7, 8 | |  |
|  | 13c | Describe any methods used to tabulate or visually display results of individual studies and syntheses. | 7, 8 | |  |
|  | 13d | Describe any methods used to synthesize results and provide a rationale for the choice(s). If meta-analysis was performed, describe the model(s), method(s) to identify the presence and extent of statistical heterogeneity, and software package(s) used. | 7, 8 | |  |
|  | 13e | Describe any methods used to explore possible causes of heterogeneity among study results (e.g. subgroup analysis, meta-regression). | 8 | |  |
|  | 13f | Describe any sensitivity analyses conducted to assess robustness of the synthesized results. | 8 | |  |
| Reporting bias assessment | 14 | Describe any methods used to assess risk of bias due to missing results in a synthesis (arising from reporting biases). | 8 | |  |
| Certainty assessment | 15 | Describe any methods used to assess certainty (or confidence) in the body of evidence for an outcome. | 7 | |  |
| **RESULTS** | | | |  | |
| Study selection | 16a | Describe the results of the search and selection process, from the number of records identified in the search to the number of studies included in the review, ideally using a flow diagram. | Appendix, 6 | |  |
|  | 16b | Cite studies that might appear to meet the inclusion criteria, but which were excluded, and explain why they were excluded. | Appendix, 6 | |  |
| Study characteristics | 17 | Cite each included study and present its characteristics. | Appendix, 3 | |  |
| Risk of bias in studies | 18 | Present assessments of risk of bias for each included study. | Appendix, 8 | |  |
| Results of individual studies | 19 | For all outcomes, present, for each study: (a) summary statistics for each group (where appropriate) and (b) an effect estimate and its precision (e.g. confidence/credible interval), ideally using structured tables or plots. | 8-11; 10-13; 22-26 | |  |
| Results of syntheses | 20a | For each synthesis, briefly summarise the characteristics and risk of bias among contributing studies. | Appendix, 6 | |  |
|  | 20b | Present results of all statistical syntheses conducted. If meta-analysis was done, present for each the summary estimate and its precision (e.g. confidence/credible interval) and measures of statistical heterogeneity. If comparing groups, describe the direction of the effect. | 10-12 | |  |
|  | 20c | Present results of all investigations of possible causes of heterogeneity among study results. | 10-12 | |  |
|  | 20d | Present results of all sensitivity analyses conducted to assess the robustness of the synthesized results. | 10-12 | |  |
| Reporting biases | 21 | Present assessments of risk of bias due to missing results (arising from reporting biases) for each synthesis assessed. | 10-12 | |  |
| Certainty of evidence | 22 | Present assessments of certainty (or confidence) in the body of evidence for each outcome assessed. | 10-12 | |  |
| **DISCUSSION** | | | |  | |
| Discussion | 23a | Provide a general interpretation of the results in the context of other evidence. | 12 | |  |
|  | 23b | Discuss any limitations of the evidence included in the review. | 15 | |  |
|  | 23c | Discuss any limitations of the review processes used. | 15 | |  |
|  | 23d | Discuss implications of the results for practice, policy, and future research. | 12-15 | |  |
| **OTHER INFORMATION** | | | |  | |
| Registration and protocol | 24a | Provide registration information for the review, including register name and registration number, or state that the review was not registered. | 8 | |  |
|  | 24b | Indicate where the review protocol can be accessed, or state that a protocol was not prepared. | 8 | |  |
|  | 24c | Describe and explain any amendments to information provided at registration or in the protocol. | N/A | |  |
| Support | 25 | Describe sources of financial or non-financial support for the review, and the role of the funders or sponsors in the review. | 17 | |  |
| Competing interests | 26 | Declare any competing interests of review authors. | 17 | |  |
| Availability of data, code and other materials | 27 | Report which of the following are publicly available and where they can be found: template data collection forms; data extracted from included studies; data used for all analyses; analytic code; any other materials used in the review. | N/A | |  |

**Search Strategy: PubMed/MEDLINE**

(((((("angioplasty, balloon, coronary"[MeSH Terms] OR ("angioplasty"[All Fields] AND "balloon"[All Fields] AND "coronary"[All Fields]) OR "coronary balloon angioplasty"[All Fields] OR ("balloon"[All Fields] AND "angioplasty"[All Fields]) OR "balloon angioplasty"[All Fields] OR "angioplasty, balloon"[MeSH Terms] OR ("angioplasty"[All Fields] AND "balloon"[All Fields]) OR "balloon angioplasty"[All Fields] OR ("balloon"[All Fields] AND "angioplasty"[All Fields])) AND (("cutting"[All Fields] OR "cuttings"[All Fields]) AND ("balloon"[All Fields] OR "balloon s"[All Fields] OR "balloons"[All Fields]))) OR (("score"[All Fields] OR "score s"[All Fields] OR "scored"[All Fields] OR "scores"[All Fields] OR "scoring"[All Fields] OR "scorings"[All Fields]) AND ("balloon"[All Fields] OR "balloon s"[All Fields] OR "balloons"[All Fields]))) AND ("calcium"[MeSH Terms] OR "calcium"[All Fields] OR "calciums"[All Fields] OR "calcium s"[All Fields])) OR (("calcifiability"[All Fields] OR "calcifiable"[All Fields] OR "calcified"[All Fields] OR "calcifier"[All Fields] OR "calcifiers"[All Fields] OR "calcifies"[All Fields] OR "calcify"[All Fields] OR "calcifying"[All Fields]) AND ("lesion"[All Fields] OR "lesion s"[All Fields] OR "lesional"[All Fields] OR "lesions"[All Fields]))) AND ("percutaneous coronary intervention"[MeSH Terms] OR ("percutaneous"[All Fields] AND "coronary"[All Fields] AND "intervention"[All Fields]) OR "percutaneous coronary intervention"[All Fields]) AND ("stent s"[All Fields] OR "stentings"[All Fields] OR "stents"[MeSH Terms] OR "stents"[All Fields] OR "stent"[All Fields] OR "stented"[All Fields] OR "stenting"[All Fields])) OR ("clinical trials as topic"[MeSH Terms] OR ("clinical"[All Fields] AND "trials"[All Fields] AND "topic"[All Fields]) OR "clinical trials as topic"[All Fields] OR "trial"[All Fields] OR "trial s"[All Fields] OR "trialed"[All Fields] OR "trialing"[All Fields] OR "trials"[All Fields]) OR (("random allocation"[MeSH Terms] OR ("random"[All Fields] AND "allocation"[All Fields]) OR "random allocation"[All Fields] OR "random"[All Fields] OR "randomization"[All Fields] OR "randomized"[All Fields] OR "randomisation"[All Fields] OR "randomisations"[All Fields] OR "randomise"[All Fields] OR "randomised"[All Fields] OR "randomising"[All Fields] OR "randomizations"[All Fields] OR "randomize"[All Fields] OR "randomizes"[All Fields] OR "randomizing"[All Fields] OR "randomness"[All Fields] OR "randoms"[All Fields]) AND ("clinical trials as topic"[MeSH Terms] OR ("clinical"[All Fields] AND "trials"[All Fields] AND "topic"[All Fields]) OR "clinical trials as topic"[All Fields] OR "trial"[All Fields] OR "trial s"[All Fields] OR "trialed"[All Fields] OR "trialing"[All Fields] OR "trials"[All Fields])).

**Supplemental Table 2. Main features of the trials included in the analysis**

|  | *Trial* | *Multicenter* |  | *Enrollment period* |  | *Comparison (randomization ratio)* | *Main inclusion criteria* | *Main exclusion*  *criteria* | *Primary endpoints* | *Available follow-up (months)* |
| --- | --- | --- | --- | --- | --- | --- | --- | --- | --- | --- |
|  | **COPS** | Yes |  | September 2019 - June 2021 |  | Cutting balloon versus non-compliant balloon (1:1) | Severely calcified de novo lesion; RVD ≥2.5 to 4.0 mm; calcium arc >100° at intracoronary imaging | N/R | MSA at calcium site and stent symmetry | 12 |
|  | **Han et al.** | No |  | January 2016 - January 2019 |  | RA plus cutting balloon versus RA plus semi-compliant balloon  (1:1) | Severely calcified de novo lesion de novo lesion, coronary Agatston calcification score >400; calcium arc >270° at intracoronary imaging | Ulcerative, thrombotic or completely occluded target lesion; left ventricular ejection fraction <30% | Success rate, differences in IVUS characteristics between the two groups and rates of residual stenosis <10% | 24 |
|  | **ISAR-CALC** | Yes |  | July 2018 - September 2019 |  | Super high-pressure non-compliant balloon versus scoring balloon  (1:1) | Severely calcified de novo lesion de novo lesion in a native coronary artery; RVD ≥2.25 to 4.00 mm; unsuccessful lesion preparation with standard non-compliant balloon | MI within 1 week; target lesion is located in a graft, is an ISR, is aorto-ostial, contains thrombus | Stent expansion index assessed by OCT | 24 |
|  | **Li et al.** | Yes |  | January 2010 - September 2014 |  | RA plus cutting balloon versus RA plus non-compliant balloon  (1:1) | Severely calcified de novo lesion; calcium arc ≥180° at intracoronary imaging | Target lesion is an ISR, is located in a graft, is extremely tortuous; coronary artery dissection; emergent percutaneous intervention | Acute lumen  gain | 12 |
|  | **PREPARE-CALC** | Yes |  | September 2014 - October 2017 |  | Scoring/cutting balloon versus RA plus non-compliant balloon  (1:1) | Severely calcified de novo lesion de novo lesion in a native coronary artery; RVD ≥2.25 to 4.00 mm | MI within 1 week; decompensated heart failure; target lesion is located in a graft, is an ISR, is aorto-ostial, contains thrombus | Strategy success and in-stent LLL at 9 months | 9 |
|  | **Tang et al.** | No |  | March 2012 - March 2013 |  | Cutting balloon versus non-compliant balloon (1:1) | Severely calcified de novo lesion; calcium arc ≥180°; calcium length ratio ≥0.5 at intracoronary imaging | Target lesion is located in a graft, is an ISR, is extremely tortuous; coronary artery dissection | Differences in IVUS characteristics between the two groups | 6 |

ISR: in-stent restenosis; IVUS: intravascular ultrasound; LLL: late lumen loss; MI: myocardial infarction; MSA: minimal stent area; N/R: not reported; OCT: optical coherence tomography; RA: rotational atherectomy; RVD: reference vessel diameter.

Official titles and acronyms: COPS: Cutting balloon to Optimize Predilation for Stent implantation; ISAR-CALC: Comparison of Strategies to Prepare Severely Calcified Coronary Lesions; PREPARE-CALC: Comparison of Strategies to Prepare Severely Calcified Coronary Lesions.

|  | *Trial* | *Major adverse cardiac events* | *Repeat revascularization* |  | *Myocardial infarction* | *Death* | *Coronary perforation* | *Minimal stent area* |
| --- | --- | --- | --- | --- | --- | --- | --- | --- |
|  | **COPS** | N/R* | Any revascularization of the target lesion |  | N/A | Death from any cause | Rupture of a coronary vessel according to Ellis et al. | Smallest cross-sectional area of the stent |
|  | **Han et al.** | New onset of severe arrhythmias, recurrent angina, recurrent heart failure, TVR, in-stent thrombosis or restenosis, non-fatal MI, cardiac death | Any revascularization of the target vessel |  | Non-fatal MI | Cardiac death | Rupture of a coronary vessel according to Ellis et al. | Smallest cross-sectional area of the stent |
|  | **ISAR-CALC** | Cardiac death, target vessel-related MI and repeat revascularisation | Any revascularization of the target vessel |  | Target vessel-related MI | Death from any cause | Rupture of a coronary vessel according to Ellis et al. | Smallest cross-sectional area of the stent |
|  | **Li et al.** | Any death, any MI,  and TVR | Any revascularization due to restenosis, either within the target lesion or within the same coronary artery |  | Third universal definition of MI | Death from any cause | Rupture of a coronary vessel according to Ellis et al. | Smallest cross-sectional area of the stent |
|  | **PREPARE-CALC** | Cardiac death, target vessel-related MI, and clinically-driven TVR | Any revascularization of the target vessel |  | Academic Research Consortium definition of MI | Death from any cause | Rupture of a coronary vessel according to Ellis et al. | Smallest cross-sectional area of the stent |
|  | **Tang et al.** | Any death, any MI,  and TVR† | Any revascularization due to restenosis, either within the target lesion or within the same coronary artery |  | Third universal definition of MI | Death from any cause | Rupture of a coronary vessel according to Ellis et al. | Smallest cross-sectional area of the stent |

**Supplemental Table 3. Endpoint definitions among trials included in the analysis**

*The definition has not been reported. †Corresponding to the definition of target vessel failure in this trial.

MI: myocardial infarction; N/A: not applicable; N/R: not reported; TVR: target vessel revascularization.

Official titles and acronyms: COPS: Cutting balloon to Optimize Predilation for Stent implantation; ISAR-CALC: Comparison of Strategies to Prepare Severely Calcified Coronary Lesions; PREPARE-CALC: Comparison of Strategies to Prepare Severely Calcified Coronary Lesions.

**Supplemental Table 4. League of risk estimates for primary outcome from network meta-analysis**

|  | ***Cutting***  ***balloon*** | ***Non-compliant***  ***balloon*** | ***RA/***  ***non-compliant***  ***balloon*** | ***RA/***  ***semi-compliant***  ***balloon*** | ***Scoring***  ***balloon*** | ***Super***  ***high-pressure***  ***balloon*** |
| --- | --- | --- | --- | --- | --- | --- |
| ***Cutting balloon*** | ― | 0.34  [0.11-1.05] | 0.51  [0.10-2.63] | 0.41  [0.17-0.99] | 0.38  [0.06-2.64] | 0.48  [0.06-3.89] |
| ***Non-compliant balloon*** | 2.95  [0.09-9.15] | ― | 1.52  [0.21-11.07] | 1.20  [0.28-5.07] | 1.14  [0.12-10.63] | 1.42  [0.13-15.32] |
| ***RA/***  ***non-compliant balloon*** | 1.94  [0.38-9.94] | 0.66  [0.09-4.79] | ― | 0.79  [0.12-5.08] | 0.75  [0.27-2.08] | 0.93  [0.25-3.45] |
| ***RA/***  ***semi-compliant balloon*** | 2.46  [1.00 -6.00] | 0.83  [0.19-3.51] | 1.26  [0.19-8.12] | ― | 0.95  [0.11-7.92] | 1.18  [0.12-11.49] |
| ***Scoring balloon*** | 2.59  [0.38-17.78] | 0.88  [0.09-8.18] | 1.33  [0.48-3.70] | 1.05  [0.13-8.81] | ― | 1.25  [0.55-2.81] |
| ***Super***  ***high-pressure balloon*** | 2.07  [0.26-16.75] | 0.70  [0.06-7.55] | 1.07  [0.28-3.93] | 0.84  [0.09-8.18] | 0.80  [0.36-1.80] | ― |

Risk estimates are reported as risk ratio [95% Confidence interval]. A risk ratio <1 means that the risk of having an event for the column therapy is lower than that for the row therapy. RA: rotational atherectomy

**SUPPLEMENTAL FIGURES LEGEND**

**S-Figure 1**: **PRISMA flow chart for the trial selection process.**

PRISMA: Preferred Reporting Items for Systematic reviews and Meta-Analyses. RCTs: randomised controlled trials

**S-Figure 2**: **Cochrane risk of bias tool for randomized trials (RoB 2).**

Official titles and acronyms: COPS: Cutting balloon to Optimize Predilation for Stent implantation; ISAR-CALC: Comparison of Strategies to Prepare Severely Calcified Coronary Lesions; PREPARE-CALC: Comparison of Strategies to Prepare Severely Calcified Coronary Lesions.

**S-Figure 3**: **Network of treatment strategies for the primary outcome with modified balloon versus control therapy**

The nodes in the graph layout correspond to the treatments and edges display the observed treatment comparisons for major adverse cardiac events. RA: rotational atherectomy.

**S-Figure 4**: **Influence analyses for main outcomes** **with modified balloon versus control therapy.**

Random-effects estimates for major adverse cardiac events associated with modified balloon versus control therapy computed omitting one study at time. The diamonds indicates the point estimate and the left and the right ends of the lines the [95% Confidence intervals]. Official titles and acronyms are as in the **S-Figure 2**.

**S-Figure 5**: **Funnel plot for the primary outcome with modified balloon versus control therapy.**

The publication bias for major adverse cardiac events is evaluated by visual inspection and by a linear regression test of funnel plot asymmetry (see text).

**SUPPLEMENTAL FIGURES**

**
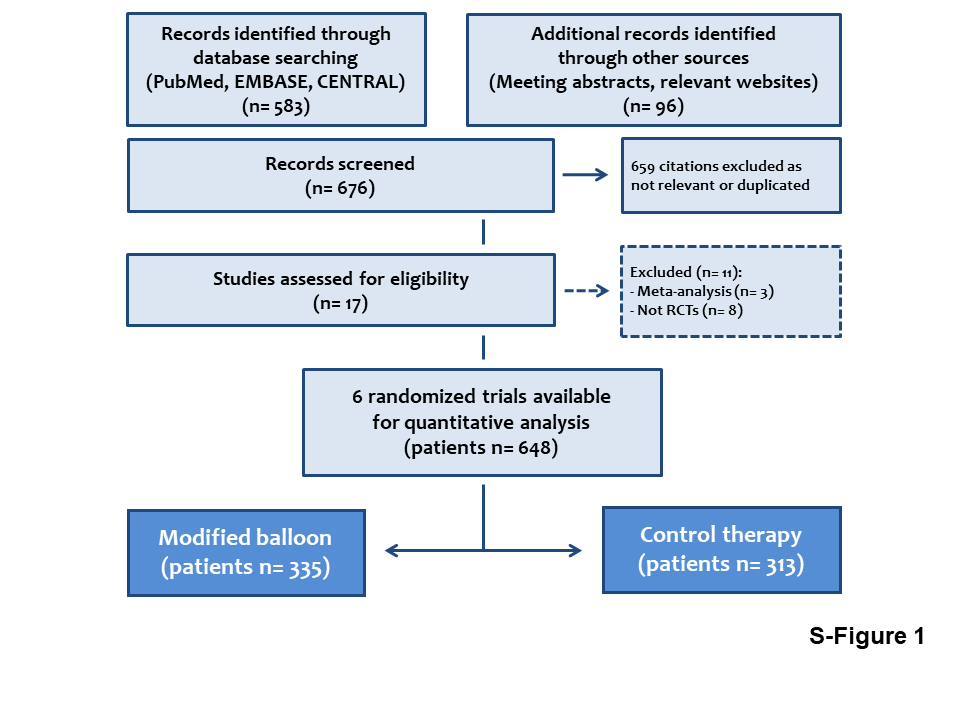

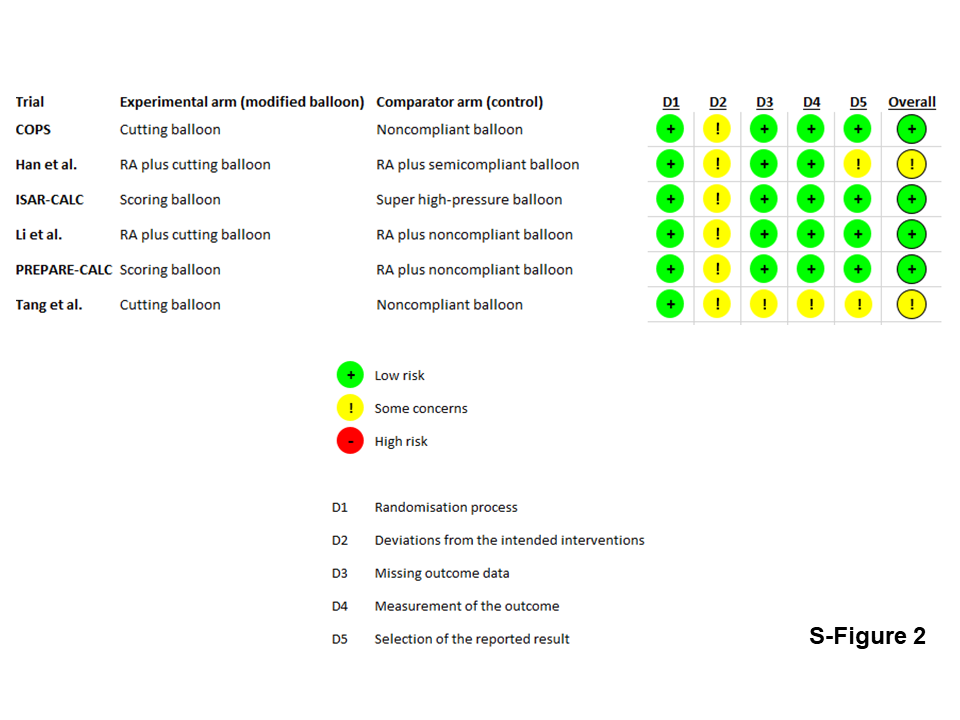

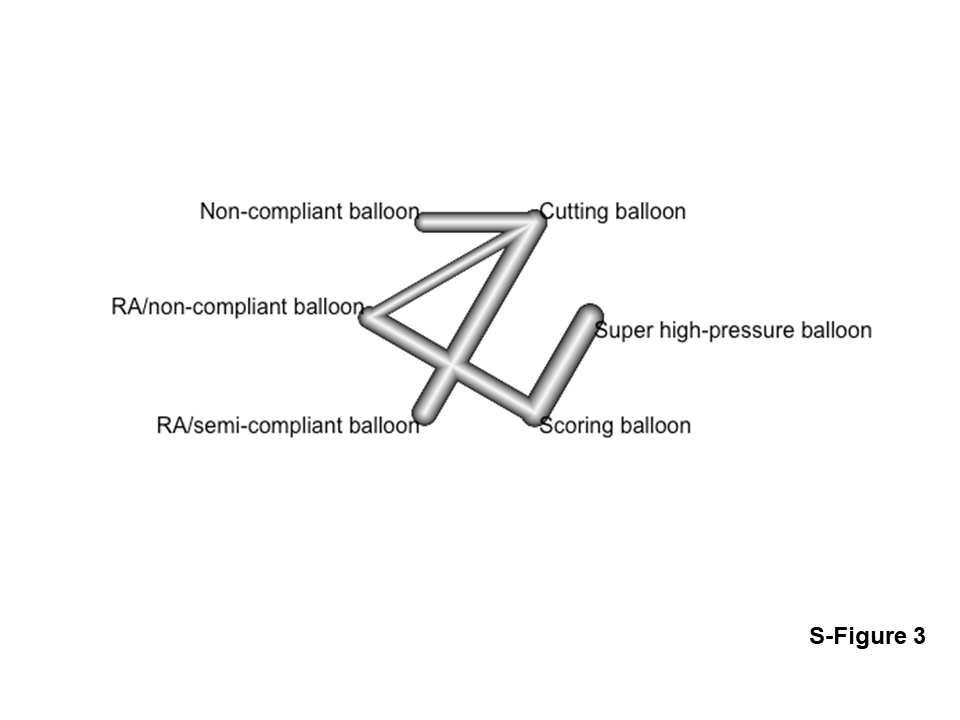

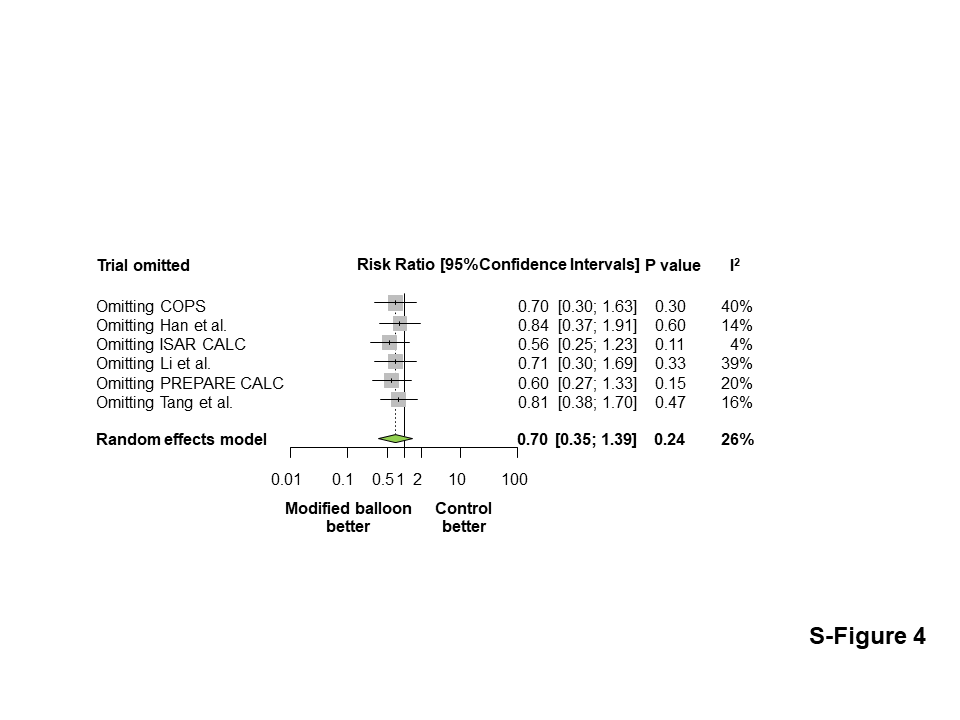

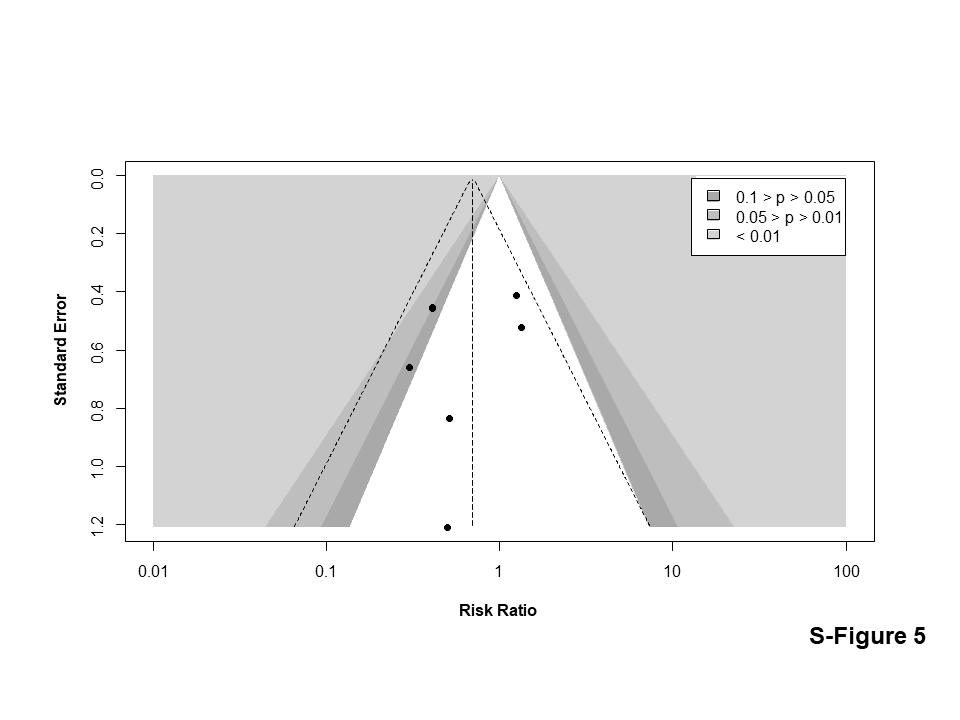
**
